# New York Medical Gazette, and Journal of Health

**Published:** 1850-08

**Authors:** 


*New York Medical Gazette, and Journal of Health.*—This is the title of a new periodical to be issued weekly by S. S. & W. Wood, of New

York, and edited by D. Meredeth Reese, M. D., LL. D. Price two dollars per annum, in advance.

It will be perceived that the work is designed for unprofessional, as well as medical readers. To meet conjointly the wants of both is a difficult undertaking, but we know of no one more likely to succeed in the attempt than Dr. Reese, and we heartily wish him success.

